# Characterization of the Complete Genome of Chikungunya in Zhejiang, China, Using a Modified Virus Discovery Method Based on cDNA-AFLP

**DOI:** 10.1371/journal.pone.0083014

**Published:** 2013-12-18

**Authors:** Yi Sun, JuYing Yan, HaiYan Mao, Lei Zhang, QinFeng Lyu, ZhongHua Wu, Wei Zheng, Cen Feng, YanJun Zhang

**Affiliations:** 1 Zhejiang Provincial Center for Disease Control and Prevention, Hangzhou, Zhejiang, P.R.China; 2 Zhejiang International Travel Healthcare Center, Hangzhou, Zhejiang, P.R.China; 3 Zhejiang Normal University, Jinhua, Zhejiang, P.R.China; University of Kansas Medical Center, United States of America

## Abstract

**Background:**

Chikungunya (CHIK) virus is a mosquito-borne emerging pathogen presenting great health challenges worldwide, particularly in tropical zones. Here we report a newly detected strain of CHIK, Zhejiang/chik-sy/2012, in China, a nonindigenous region for CHIK, using a modified approach based on the classic cDNA-AFLP. We then performed etiological and phylogenetic analyses to better understand its molecular characterization and phylogenetic pattern, and also to aid in further evaluating its persistence in Southeast Asia.

**Methods:**

By using this modified procedure, we determined for the first time the complete genome sequence of the chikungunya virus strain, Zhejiang/chik-sy/2012, isolated in 2012 from a patient in Zhejiang, China. Sequence analyses revealed that this positive single strand of RNA is 12,017 bp long. We found no single amino acid mutation in A226V, D284E and A316V. Phylogenetic analysis showed that our strain shared the greatest homology with a strain isolated in Taiwan, which was derived from a strain from Indonesia. Chik-sy/2012 is in a different clade from other CHIK viruses found in China previously.

**Conclusions:**

A modified cDNA-AFLP in virus discovery was used to isolate the first CHIK and the first complete genome sequence of virus strain chik-sy/2012 in 2012 from a patient with CHIK fever in Zhejiang, China. The infection displayed great phylogenetic distance from viruses detected in Guangdong, China, in 2008 and 2010, since they were derived from another evolutionary lineage. Additional molecular epidemiology data are needed to further understand, monitor and evaluate CHIK in China.

## Introduction

Chikungunya (CHIK), an *Alphavirus* in the family *Togaviridae*, causes human febrile illness accompanied by severe, chronic joint pain. It has emerged and reemerged in Africa and Asia since the mid 1950s and exploded onto the global scene as a major emerging pathogen in a series of devastating outbreaks since 2004 [1], [2]. CHIK is a potential global threat to public health because it is transmitted by mosquitoes in areas where there are large populations of susceptible human hosts. Phylogenetic and phylogeographic analyses showed that CHIK of African origin spread by two distinct routes, one throughout the Indian Ocean and the other moving from India to scattered locations in Southeast Asia and then to Italy [1]. The first incidence of CHIK in mainland China was firstly detected in 2008 [3]. A sudden outbreak among 173 patients was also reported in Guangdong Province, China, in 2010 [4]. In this article, we report the first isolation of CHIK virus in Zhejiang province, China, a nonindigenous region for CHIK, using a modified approach based on the classic cDNA-AFLP in virus discovery. We propose this modification as a better technique because it may improve accuracy in the hunt for viral segments and in determining the genome sequence of both DNA and RNA viruses on a larger scale. Our aims in this study were therefore the following: 1) test our modified work flow of the classic cDNA-AFLP method using CHIK isolation as the study material; 2) determine the entire genome sequence of CHIK; 3) understand the molecular characterization of the CHIK isolate and the phylogenetic patterns of CHIK in Asia to aid in further studies and to evaluate its persistence in China.

## Materials and Methods

### Isolation of an unknown pathogen

A 200 µl serum sample from a suspected case of CHIK in Zhejiang Province, China, was obtained from the Zhejiang Entry-Exit Inspection and Quarantine Bureau in July 2012. Because the cause of the illness was unknown, we designed a strategy to isolate the pathogen by inoculating multiple cell lines susceptible to viral agents, including Vero, C6/36 and BHK-21. The cell lines we used are gifts from National Institute for Viral Disease Control and Prevention. The cells were cultured at 37°C, 28°C and 37°C in a 5% carbon dioxide atmosphere with media changes twice a week. The cultures were checked daily for cytopathic effects (CPEs).

### Genetic Analysis

**Figure 1:**
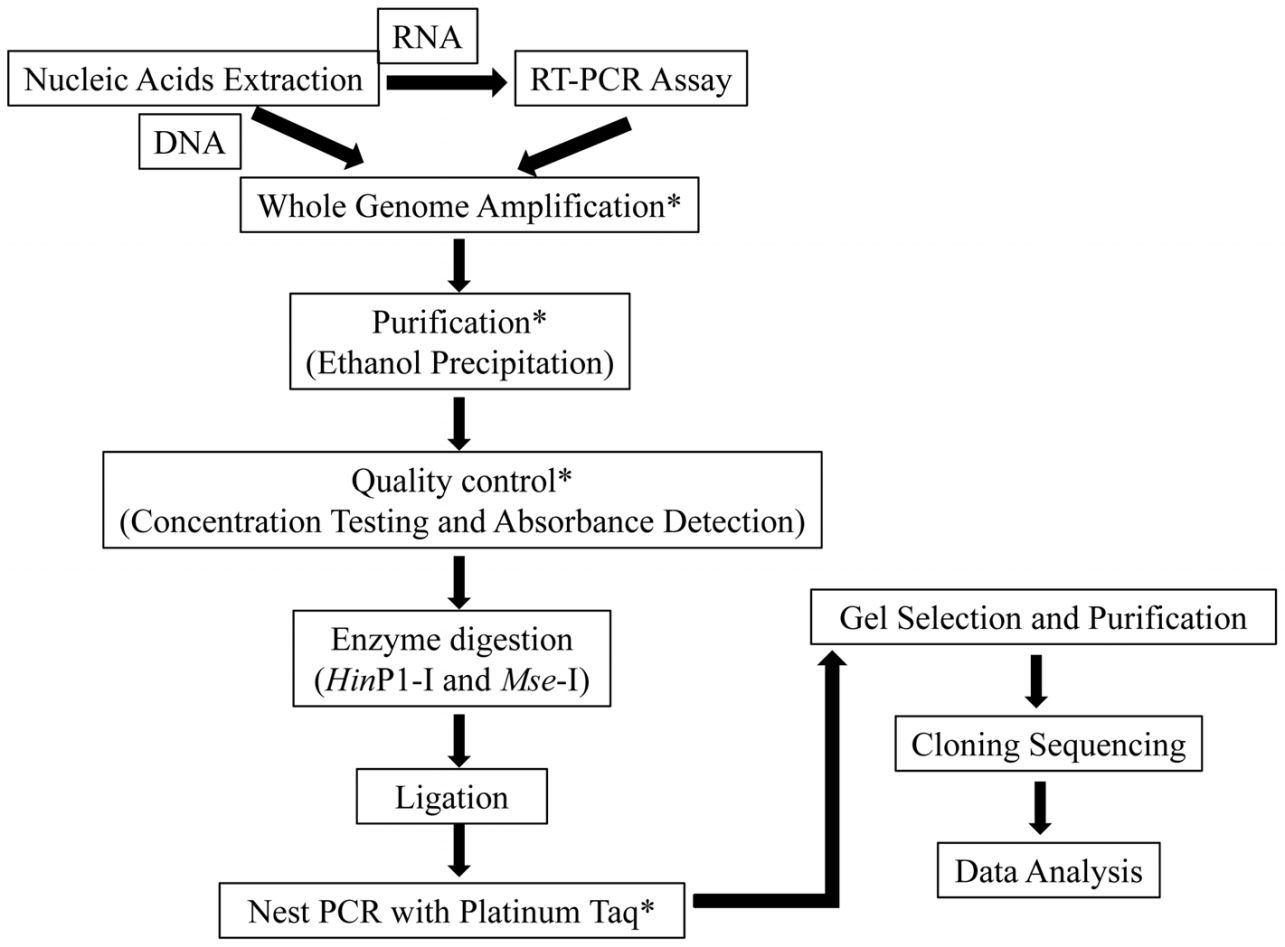
Full work flow of our modified approach based on the classic cDNA-AFLP for discovering viruses. Modification steps are indicated with asterisks.

For the CHIK isolate, the viral RNA was first extracted using the RNeasy Mini Kit (Qiagen) according to the manufacturer's instructions. The RT-PCT assay was conducted using the Revertaid First Strand cDNA Synthesis Kit (Fermentas). The classic cDNA-AFLP method was enzyme digestion, followed by ligation, nest PCR and clone sequencing [5]. Our modified workflow started with whole genome amplification (WGA) using QIAGEN REPL-g Mini Kit (Qiagen). We applied ethanol precipitation as purification for the post-WGA procedure. We used NanoVue (GE Healthcare) for quality control, including concentration testing, and absorbance detection. We then performed enzyme digestion using *Hin*P1-I and *Mse*-I for 2 h at 37°C. Adaptor ligation was followed using the pre-mixed adaptors (*Hin*P1-I anchors. Top strand: 5-GACGATGAGTCCTGA C-3; Bottom strand: 5-CGGTCAGGACTCAT-3; *Mse*-I anchor: Top strand: 5-CTCGTAGACTGCGTACC-3; Bottom strand: 5-TAGGTACGCAGTC-3). The ligation was performed for 2 h at 37°C. Based on nested PCR according to classic methods [5], we applied different DNA polymerases. We chose Platinum Taq DNA polymerase high fidelity (Life Technologies) instead of AmpliTag Gold (Life Technologies), as the former enzyme has improved sensitivity and its amplicon size can be up to 8 kb. The system of nested PCR was adjusted following the manufacturer's instructions according to the changed enzyme. Primer sets for whole nest PCR are in table 1. According to the classic cDNA-AFLP methods, the first PCR run was initial denaturing for 5 min at 94°C followed by 20 cycles of 1 min at 94°C, 1 min at 55°C, 2 min of elongation at 72°C, ending with a 10-min extension at 72°C. The second PCR run started with denaturing for 5 min at 94°C, followed by 10 cycles of 1 min at 94°C, 1 min at 65 to 56°C with −1°C per cycle for each successive cycle and 1 min 30 s of elongation at 72°C; then started again with 23 cycles of 30 s at 94°C, 30 s at 56°C, 1 min at 72°C and 10 min 72°C elongation [5]. The second PCR product was analyzed on agarose gel. DNA fragments from the gel were extracted with the QIAquick get extraction kit (Qiagen) following the manufacturer's protocol. Finally, we performed clone sequencing. The full workflow is shown in Figure 1. Using Geneious v4.8.3 (www.geneious.com) we calibrated the fragment sequences manually and blasted them online. With the information obtained, and knowing the position of each fragment, we designed 12 pairs of primers using Primer Premier 5.0 (PREMIER Biosoft International) to determine the whole genome of CHIK (Table 2). The 5′ terminals of the viral RNA segments were determined with a RACE Kit (Life Technologies). The whole genome of CHIK was assembled and aligned with 154 additional sequences downloaded from GenBank by Geneious. Dataset-specific models that were selected using the Akaike Information Criterion in Modeltest 3.7 were analyzed. Maximum likelihood (ML) analysis was processed in RAxML v7.2.8 [6], [7]. The optimal ML tree and bootstrap percentages (BP) were estimated in the same run. The ML BP values were obtained from 1000 bootstrap replicates using the rapid bootstrap algorithm.

**Table 1:** Sequences of two primer set in nested PCR.

Run	*Hin*P1-I	*Mse-*I
First run	5-GACGATGAGTCCTGACCGC-3	5-CTCGTAGACTGCGTACCTAA-3
Second run	5-GACGATGAGTCCTGACCGCA-3	5-CTCGTAGACTGCGTACCTAAA-3
	5-GACGATGAGTCCTGACCGCT-3	5-CTCGTAGACTGCGTACCTAAT-3
	5-GACGATGAGTCCTGACCGCC-3	5-CTCGTAGACTGCGTACCTAAC-3
	5-GACGATGAGTCCTGACCGCG-3	5-CTCGTAGACTGCGTACCTAAG-3

**Table 2:** Characteristics of 12 primers developed for chikungunya.

Locus	Primer sequences (5′-3′)	Ta (°C)
1	F: CAGCAAGGAGGATGATGTCG	59
	R: CGTCTTCACTTGCTCCGCTA	
2	F: ACTGCCCAACTAACAGACCA	55
	R: GCCTAACTGCGTAAACTCCT	
3	F: GTGCTTCAGAGGGTGGGTTA	58
	R: TTTGCGGTTCCTACTGGTGT	
4	F: ACGATGAAGAGTGCGTGGTC	56
	R:GTAGGAAGTCTCCGAAAGTTAGT	
5	F: GGCACCGCCAAGTATCAC	56
	R: CTCCCAAATCTTCTAACAGC	
6	F: AGATTGAACGCCGTCCTC	55
	R: CCGTGGTGCCAGTTGTAG	
7	F: GGGGACAAAGTAATGAAGCC	57
	R: TTGCCAGAGGAAATGGAATG	
8	F: GCGGTCACCAATCACAAA	55
	R: TAAAGGCTGCTGCTCGTT	
9	F: GGGAGAAGAACCAAACTATCA	54
	R: GACGCTCAGTACGGCTAAA	
10	F: GGAACGAGCAGCAGCCTTTA	61
	R: CAGCGACAACCAGTCCCACA	
11	F: CGGTAAGAGCGATGAACTGC	65
	R: TGGGTACGGAGAATTGTGGA	
12	F: GGTGCTATGCGTGTCGT	52
	R:GAATATT(A)14	

Forward (F) and reverse (R) primer sequences and optimal annealing temperatures (Ta) are shown for each primer.

## Results

### Virus Isolation

**Figure 2:**
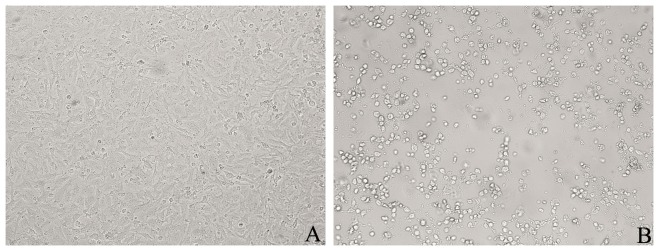
Virus-induced cellular changes visible by ×100 light microscopy in Vero cells. Panel A shows vero cell in normal condition. Panel B shows cytopathic effect (CPE) of cells infected with CHIK 3 days after inoculation in vero.

This was the first suspected case of CHIK in Zhejiang Province. Three days after inoculation, virus-induced cellular changes were observed using light microscopy in two cells lines, Vero and C6/36. Both cell lines showed significant cytopathic effects, including small, round cells, some of which were damaged. Granular particles were found in the cytoplasm (Figure 2). BHK-21 cells showed CPE after six days of inoculation. After several passes in culture, the CPE usually appeared 3 or 4 days after inoculation of a fresh monolayer. There were no significantly different CPEs among three cell lines.

### Sample concentration comparison

Targeted sample concentration usually plays a crucial role in such experiments. To improve the final targeted fragments concentration, we added several steps to our modified workflow. WGA followed with ethanol precipitation is one of the main procedures, which we repeated for two independent runs. Table 3 shows the comparison of sample concentration before and after whole genome amplification between two runs. Ratios between post- and pre-WGA were 190 and 138 respectively, which suggests that the WGA method could greatly improve the concentration of sample nucleotides. As a result, thirty four sequences (79.07%) out of 43 clones chosen at random were identified as CHIK fragments, proving that this modified workflow for virus discovery is feasible and reliable.

**Table 3:** Comparison of sample concentration before and after whole genome amplification (WGA).

Concentration	First run	Second run
Before WGA (ng/µl)	28	29
After WGA (ng/µl)	5320	3988
Ratio	190	138

### Molecular characterization

Using the modified method above, we determined the complete genome of the CHIK pathogen in Zhejiang province (GenBank accession number: KF318729). The positive single strand of RNA was 12,017 bp long with three parts: a 5′ noncoding area 76 bp long, a 3′ noncoding area 716 bp long including an I-poly A region, and the coding area. The calculated base composition was: A (29.6%), C (24.3%), G (25.3%) and T (20.8). Two independent open reading frames (ORFs) were embedded: one, from 77 bp to 7489 bp, encodes the non-structural protein nsP1 to nsp4. The other, from 7555 to 11301 bp, codes structural proteins, such as C, E3, E2, 6K and E1. The untranslated junction region between the two coding areas was 66 bp long. The sequence structure was therefore 5′ cap - nsP1 - nsP2 – nsP3 - nsP4 - junction region – C - E3 – E2 - 6K - E1 - poly A -3.′

We compared an 11159 bp coding region from our isolate chik-sy/2012 and 16 other complete genomic sequences detected in China since 2008 with 22 other whole genome sequences taken from CHIKs isolated during different years (Table 4) [3]. Amino acid differences were found in all genes; the most variable genome regions were in the structural proteins. No specific amino acid changes were found in chik-sy/2012 including A226V, D284E and A316V.

**Table 4:** Unique amino acid changes observed in 17 CHIVs detected in China compared to 22 earlier published strains*.

	Non-structural proteins							Structural proteins							
	nsP1	nsP2	nsP2	nsP2	nsP3	nsP3	nsP4	C	E2	E2	6K	E1	E1	E1	E1
Polypeptide position	120	1074	1112	1167	1705	1727	2044	8	503	577	779	1035	1078	1093	1125
Protein position	Q120R	L539S	G577R	N632S	D372N	M394I	P181S	T8A	R178H	K252Q	V31I	A226V	M269V	D284E	A316V
FD080008/2008	Q	L	G	N	D	I	P	T	H	K	I	A	V	E	A
SD08Pan/2008	R	L	R	S	N	M	P	T	R	K	V	A	M	E	A
FD080178/2008	Q	S	G	N	D	M	S	A	R	Q	V	V	V	E	V
FD080231/2008	Q	S	G	N	D	M	P	A	R	Q	V	V	V	E	A
chik-sy/2012	Q	L	G	N	D	M	P	T	R	K	V	A	M	D	A
GD115/2010	Q	S	G	N	D	M	P	T	R	Q	V	V	V	E	A
GD113/2010	Q	S	G	N	D	M	P	T	R	Q	V	V	V	E	A
GD139/2010	Q	S	G	N	D	M	P	T	R	Q	V	V	V	E	A
GD134/2010	Q	S	G	N	D	M	P	T	R	Q	V	V	V	E	A
DG891/2010	Q	S	G	N	D	M	P	T	R	Q	V	V	V	E	A
DG892/2010	Q	S	G	N	D	M	P	T	R	Q	V	V	V	E	A
DG893/2010	Q	S	G	N	D	M	P	T	R	Q	V	V	V	E	A
DG894/2010	Q	S	G	N	D	M	P	T	R	Q	V	V	V	E	A
DG895/2010	Q	S	G	N	D	M	P	T	R	Q	V	V	V	E	A
GZ0991/2010	Q	L	G	N	D	M	P	T	R	K	V	A	V	E	A
GZ1029/2010	Q	L	G	N	D	M	P	T	R	K	V	A	V	E	A
CHI2010/2010	Q	L	G	N	D	M	P	T	R	K	V	A	V	D	A

*Twenty-two CHIKV whole-genome sequences were selected for comparison with the imported CHIKVs, including three east/central/south African strains: S27 (GenBank: AF369024), NC004162 (GenBank: NC_004162), and ROSS (GenBank: AF490259); thirteen Indian Ocean strains: IND-00-MH4 (GenBank: EF027139), IND-06-TN1(GenBank: EF027138), CHIK31 (GenBank: EU564335), TM25 (GenBank: EU564334), DRDE-06 (GenBank: EF210157), ITA07-RA1 (GenBank: EU244823), Wuerzburg (GenBank: EU037962), D570/06 (GenBank: EF012359), IND-06-RJ1 (GenBank: EF027137), IND-06-MH2 (GenBank: EF027136), IND-06-KA15 (GenBank: EF027135), IND-06-AP3 (GenBank: EF027134), and LR2006 OPY1 (GenBank: DQ443544); five Asian strains: IND-60-WB1 (GenBank: EF027140), IND-73-MH5 (GenBank: EF027141), AF15561 (GenBank: EF452493), TSI-GSD-218 (GenBank: L37661), and MY002IMR/06/BP (GenBank: EU703759); and one West African strain: 37997 (GenBank: AY726732) [3].

**Figure 3:**
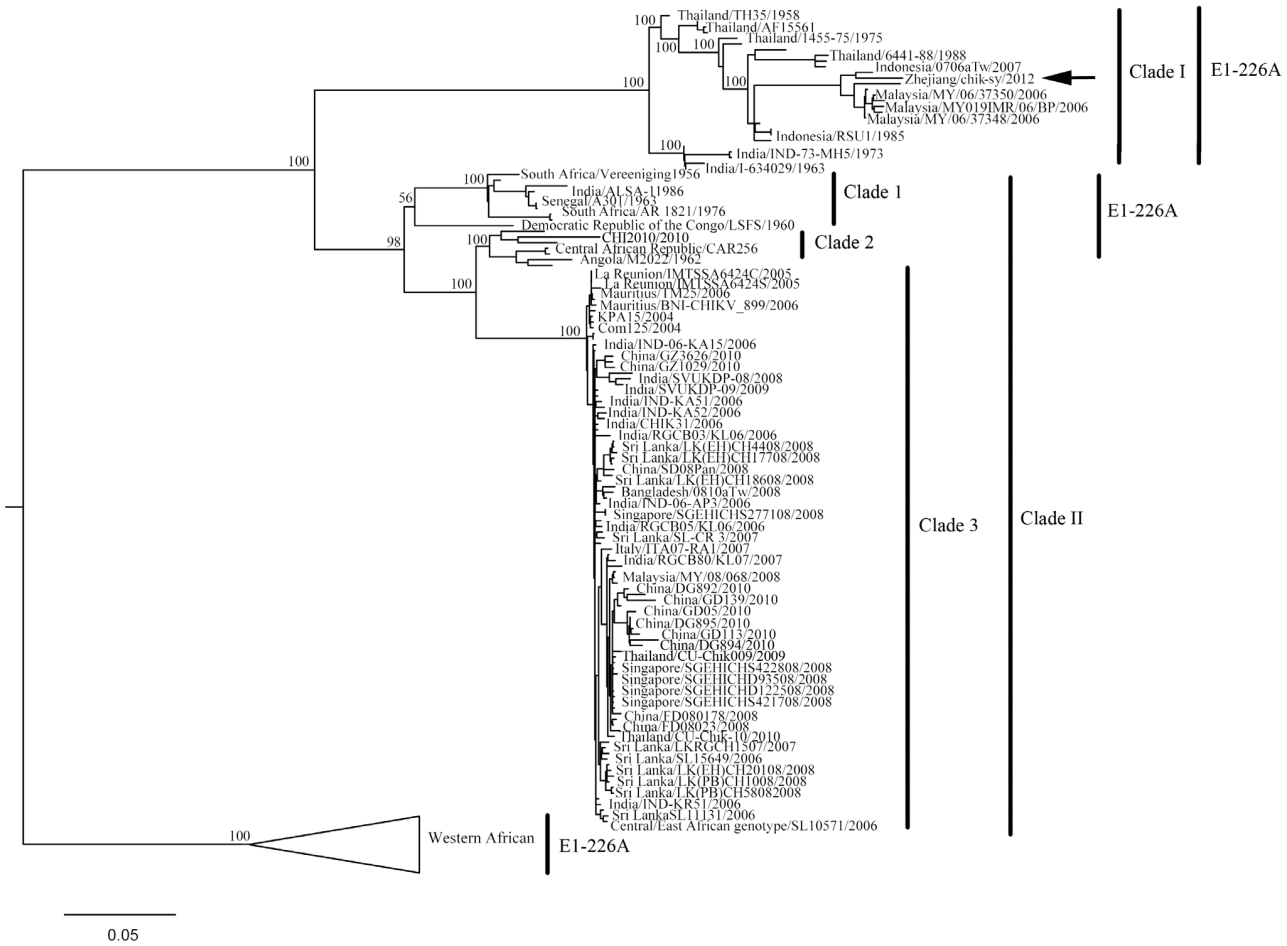
Maximum likelihood tree of chikungunya obtained by analysis of 155 sequences. Bootstrap values based on 1000 replicates are indicated above branches.

In our maximum likelihood tree with bootstrap percentage assigned, sequences from western African and other isolates were reciprocally monophyletic with clades receiving both 100% bootstrap support (Figure 3). Two clades were within the non-western African branch. Clade I was consisted of isolates from India, Thailand, Malaysia and Indonesia with E1-226A present in all sequences. Our chik-sy/2012 shared its highest identity with Indonesia/0706aTw/2007. Clade II had a more complex phylogenetic pattern, as it split into two sub-clades: clade 1 and a clade that diverged into clade 2 and 3. Isolates in clade 1 and 2 possessed 226A in the E1 gene while clade 3 had sequences derived from different areas. Some of them, such as China/GD892/2010, presented variation of A226V (Figure 3 and Table 4). The strain detected in Zhejiang province was distributed in a different clade from other CHIK viruses found in China previously (clade I and clade 3 inside clade II).

## Discussion

### Phylogeny of the chikungunya virus

This is the first isolation of CHIK and the first complete genome sequence of virus strain chik-sy/2012 isolated in 2012 from a patient with CHIK fever in Zhejiang, China. A report from the Zhejiang Entry-Exit Inspection and Quarantine Bureau showed the patient to be a sailor who traveled around Southeast Asia, such as to Indonesia and Malaysia. Our findings are consistent with the results shown in the phylogenetic tree, which this isolate shares its greatest identity with Indonesia/0706aTw/2007, a strain imported to Taiwan from Indonesia. The Zhejiang isolate was distributed inside the Southeast Asian clade that was initially spread from India (Figure 3). Because it was an imported case, transmission of the virus carried by the travelers was monitored. We believe that the infections originated in Indonesia or Malaysia rather than in China. No other local close contacts were found to be infected. Our ML tree indicated that this infection was phylogenetically greatly distant from the viruses detected in Guangdong, China, in 2008 and 2010, as they were derived from another CHIK lineage.

Previous studies hypothesized African origin of CHIK, which then migrated via the Indian area into Southeast Asia and to other parts of the world [1], [8]. From the ML tree we produced, we inferred a similar phylogenetic pattern for CHIK. CHIK has likely been circulating for years in Africa since its origin. It then diverged along two different routes: one to Southeast Asia (Thailand, Indonesia and Malaysia) from the 1950s to 1980s where it became locally endemic (Clade I); the other (Clade II) through the Indian ocean, such as Sri Lanka and La Reunion, to Southeast Asia and then to Europe (Italy), where it caused major CHIK outbreaks in the past few years [8], [9], [10], [11], [12].

There was no mutation from Ala to Val in the E1 226 position in our Zhejiang strain, while there were many A226V in E1 glycoprotein isolates in clade 3, the Indian Ocean lineage. Some strains previously detected in China during 2008 to 2010 showed an alanine-to-valine substitution in E1 the gene [3], [9]. This variant was first identified on La Reunion (IMTSSA6424C/2005). The region was known to be involved in viral entry via fusion with endosomal membranes [2], [10], [11]. Viruses that carry substitutions may be able to better adapt to the vector, *Aedes albopictus*, another species of mosquito [1], [2], [12]. Whether the A226V variants followed the same migration path, however, remains uncertain, since it was the only genotype observed during the first outbreak on La Reunion from March to June, 2005, and in Kerala and other Indian regions in 2006 [10], [11]. As CHIK has developed several genetic lineages throughout the African-Asian region, local outbreaks of sporadic strains have arisen according to this geographic pattern [1]. Adaptation of RNA viruses to a new host or vector species often results in the emergence of new viral lineages. Relative genetic variation and isolation allows local strains to evolve independently and to maintain unique genes/alleles as is required for genetic adaptation to local environments under natural selection [13]. As more imported viruses emerge and are detected in China, more detailed molecular epidemiology data are needed to further understand, monitor and evaluate the threats to this region.

### A modified workflow using cDNA-AFLP for virus discovery

There are a variety of diseases caused by pathogenic microorganisms that challenge our health, some of them newly evolved and unknown to date. Identification of these microorganisms is of vital importance in the field of medical care. Several methods have been developed to meet this demand, but disadvantages and drawbacks of the available techniques are evident when new and unknown viruses are encountered [5]. Universal PCR primers, for example, may limit the possibility of identifying a member of an unexpected family. Random priming PCR assays may fail in the face of serious sample contamination. Disadvantage of representational difference analysis is that it needs a negative control tissue from the same source from where the diseased tissue was obtained. The costs for virus detection using new generation sequencing methods are still much higher than traditional procedures. Classical virus discovery based on cDNA-AFLP methods was developed and has proved to be a general, simple and easy to use method for detecting viruses on a large scale [14], [15], [16]. A novel human coronavirus was first identified using this classical cDNA-AFLP method [15]. In this study, we therefore modified the classic technique in an attempt to improve accuracy in the hunt for viral segments and to determine the entire genome sequence of viruses. Our reworked modified method involves two steps: sample pre-processing, including WGA and ethanol precipitation for purification; and library construction using Platinum Taq for substitutions. We added quality control steps, such as concentration testing and absorbance detection, to insure sample concentration as well. In some circumstances, this modified method is suitable for determining an unrecognized viral agent in samples from patients of great medical interest or novel viral pathogens. To remove the cells, cell debris and insoluble particles such as mucus, clinical samples should be centrifuged and filtrated with filter membrane. Clinical samples will be tested though our modified virus discovery method in the next study.
